# Prevalence of Celiac Disease in Patients With Turner Syndrome: Systematic Review and Meta-Analysis

**DOI:** 10.3389/fmed.2021.674896

**Published:** 2021-06-17

**Authors:** Ghada S. M. Al-Bluwi, Asma H. AlNababteh, Linda Östlundh, Saif Al-Shamsi, Rami H. Al-Rifai

**Affiliations:** ^1^Department of Internal Medicine, College of Medicine and Health Sciences, United Arab Emirates University, Al-Ain, United Arab Emirates; ^2^Institute of Public Health, College of Medicine and Health Sciences, United Arab Emirates University, Al-Ain, United Arab Emirates; ^3^National Medical Library, College of Medicine and Health Sciences, United Arab Emirates University, Al-Ain, United Arab Emirates

**Keywords:** celiac disease, Turner syndrome, systematic review, weighted prevalence, meta-analysis

## Abstract

**Introduction:** Celiac disease (CD) is a multifactorial autoimmune disorder, and studies have reported that patients with Turner syndrome (TS) are at risk for CD. This systematic review and meta-analysis aimed to quantify the weighted prevalence of CD among patients with TS and determine the weighted strength of association between TS and CD.

**Methods:** Studies published between January 1991 and December 2019 were retrieved from four electronic databases: PubMed, Scopus, Web of Science, and Embase. Eligible studies were identified and relevant data were extracted by two independent reviewers following specific eligibility criteria and a data extraction plan. Using the random-effects model, the pooled, overall and subgroup CD prevalence rates were determined, and sources of heterogeneity were investigated using meta-regression.

**Results:** Among a total of 1,116 screened citations, 36 eligible studies were included in the quantitative synthesis. Nearly two-thirds of the studies (61.1%) were from European countries. Of the 6,291 patients with TS who were tested for CD, 241 were diagnosed with CD, with a crude CD prevalence of 3.8%. The highest and lowest CD prevalence rates of 20.0 and 0.0% were reported in Sweden and Germany, respectively. The estimated overall weighted CD prevalence was 4.5% (95% confidence interval [CI], 3.3–5.9, *I*^2^, 67.4%). The weighted serology-based CD prevalence in patients with TS (3.4%, 95% CI, 1.0–6.6) was similar to the weighted biopsy-based CD prevalence (4.8%; 95% CI, 3.4–6.5). The strength of association between TS and CD was estimated in only four studies (odds ratio 18.1, 95% CI, 1.82–180; odds ratio 4.34, 95% CI, 1.48–12.75; rate ratio 14, 95% CI, 1.48–12.75; rate ratio 42.5, 95% CI, 12.4–144.8). Given the lack of uniformity in the type of reported measures of association and study design, producing a weighted effect measure to evaluate the strength of association between TS and CD was unfeasible.

**Conclusion:** Nearly 1 in every 22 patients with TS had CD. Regular screening for CD in patients with TS might facilitate early diagnosis and therapeutic management to prevent adverse effects of CD such as being underweight and osteoporosis.

## Introduction

Celiac disease (CD), also known as celiac sprue and gluten-sensitive enteropathy, is a multifactorial autoimmune disorder arising from the interaction of diverse genetic and environmental factors (1, 2). In patients with CD, the consumption of gluten-containing grains such as wheat, barley, and rye leads to an inappropriate adaptive immune response (3, 4). Although several genes have been reported to contribute to the predisposition to CD, more than 90% of patients with CD carry the HLA-DQ2 or HLA-DQ8 haplotypes (5). Gliadin consumption or repeated gastrointestinal infections in early life in genetically predisposed individuals are considered to trigger and regulate the induction of intraepithelial lymphocytes in the small intestines, leading to villous atrophy (6–8). In turn, histological changes leading to CD result in a variety of clinical manifestations. In adults, the classical clinical manifestations include chronic diarrhea, unintentional weight loss, constipation, malabsorption, and iron deficiency anemia (9). However, 50% of patients with CD present with nonclassical or atypical signs and symptoms, such as anemia, abdominal pain, osteoporosis, osteomalacia, short stature, lymphoma, liver disease, and neurological and psychological symptoms (10, 11). In pediatric patients, CD may present with unexplained growth failure, delayed puberty, chronic diarrhea, and anemia (12) and increases the risk of depression, anxiety, eating disorders, autistic spectrum disorder, and attention-deficit/hyperactivity disorder (13).

Globally, the estimated population-based prevalence of CD is approximately 1% (14). The prevalence of CD ranges from 0.8% in Europe and Oceania to 4.0% in Africa (15). The considerable increase in the prevalence of CD worldwide observed in recent decades (16, 17) has been mainly due to the increased availability of screening tests with improved sensitivity and specificity (18–20). According to current guidelines, screening for CD is not recommended for the general population but is recommended for specific patient groups who are considered at high risk for CD (21, 22), such as relatives of patients with CD as well as patients with insulin-dependent diabetes; autoimmune thyroid disease; selective IgA deficiency; and genetic disorders, including Down syndrome, Williams syndrome, and Turner syndrome (TS) (12, 23, 24).

TS is a female genetic disorder involving the X chromosome. Typical phenotypic characteristics of TS include short stature and gonadal dysgenesis (25). Female patients with TS are at high risk of developing autoimmune diseases approximately twice as high as in the general female populations (26). An increased risk of autoimmune diseases including type 1 diabetes mellitus, thyroid disease (27, 28), and CD has been reported in patients with TS (29). According to the guidelines of the North American Society for Pediatric Gastroenterology, Hepatology and Nutrition “NASPHGAN” (30), guidelines of the European Society Pediatric Gastroenterology, Hepatology and Nutrition “ESPHGAN” (31), and the guidelines and recommendation of the TS Consensus Group (32, 33), patients with TS are recommended to be screened for CD and other autoimmune disorders. On the other hand, the latest recommendation statement of the US Preventive Services Task Force (34) concludes that the current evidence is insufficient to assess the balance of benefits and harms of screening for CD in asymptomatic persons including patients who are at increased risk of developing CD such as patients with TS (34). No systematic review to date has evaluated CD in patients with TS. The aim of the present systematic review and meta-analysis was to evaluate the existing literature and provide comprehensive quantitative evidence on the prevalence of CD among patients with TS and on the strength of association between TS and CD.

## Methods

This systematic review was conducted and reported following the 2009 Preferred Reporting Items for Systematic Review and Meta-Analysis guidelines (35) (Supplementary Table 1). The review followed a previously published protocol (36) that was also registered in PROSPERO (registration number, CRD42019131881). The published protocol was designed to estimate the strength of association between TS and CD. However, given the lack of sufficient and consistent quantitative effect measures, quantifying a pooled weighted measure of effect was unfeasible. Therefore, following the same protocol and search strategy, the present systematic review was slightly modified to quantify the weighted prevalence of CD among patients with TS. To adjust for the change, necessary minor amendments, including the extraction of information on the prevalence estimates, were implemented.

### Search Strategy

A comprehensive strategy was designed to search four electronic databases: PubMed, Scopus, Web of Science, and Embase. The search string was developed by an expert librarian (LÖ) and is available in the published protocol (36) and in Supplementary Box 1, which contains the results and search details for all databases. The literature search was performed in December 2019 with no restrictions on language or region. A publication year filter to encompass the period from January 1991 until the search date was applied. The year 1990 was defined as the start year for the present study based on the publication of the first modern guidelines for CD diagnosis by the European Society of Gastroenterology, Hepatology, and Nutrition in the same year (37). All records identified in the search were imported to Covidence systematic review software (38), where automatic de-duplication was performed and the references were prepared for blinded screening. A hand search of bibliographies of studies that were deemed eligible and previously published reviews was also performed.

### Eligibility Criteria

All observational studies, abstracts, and conference papers were considered. To be deemed eligible, an observational study had to provide quantitative or quantifiable information on the prevalence of CD and/or effect measure on the association between TS and CD regardless of the age of patients with TS screened for CD. Further information on the inclusion and exclusion criteria is available in the published protocol (36).

### Study Selection and Data Extraction

Following the predesigned eligibility criteria, titles and abstracts of the retrieved studies were independently screened by two reviewers (GSM-AB and AH-N) to identify fully as well as potentially eligible studies; the full texts of the identified studies were retrieved and thoroughly assessed for their eligibility. Conflicts between the reviewers were discussed with a third reviewer (RH-A) and resolved by consensus.

Relevant data were extracted from the studies that were deemed eligible. Data extraction was independently performed by two reviewers (GSM-AB and AH-N) following predefined data extraction parameters described in the published protocol (36), with minor amendments to extract data related to prevalence estimates. Discrepancies between the reviewers were discussed with a third reviewer (RH-A) and resolved by consensus. The following information was extracted from eligible studies: author names; publication year; country and city where the study was conducted; study design, setting, and period; CD diagnostic method; type of TS; number of participants tested for CD; mean or range of age of study participants at the time of CD testing; number of participants who were diagnosed with CD; number of patients with and without TS diagnosed with CD; and crude and adjusted estimates of the association between TS and CD with 95% confidence intervals (CIs), if available. The corresponding authors of the eligible articles were contacted by e-mail if the published information in the article was not sufficient.

### Quantitative Evidence Synthesis and Data Analysis

According to our previously published protocol (36), we aimed to estimate the strength of association between TS and CD. However, due to the lack of sufficient studies reporting estimates on the strength of association between the exposure–outcome pair, we aimed to determine the burden of CD, in the form of weighted prevalence, among patients with TS.

Among the patients with TS tested for CD, the weighted CD prevalence and corresponding 95% CI was estimated using the Dersimonian–Laird random-effects model. In the meta-analysis, to estimate the weighted prevalence, variances in the prevalence measures were stabilized using the Freeman–Tukey double arcsine transformation method (39, 40). Measures of heterogeneity, Cochran's *Q* statistic, inconsistency I-squared (*1*^2^) index, and 95% prediction interval, which estimates the 95% interval in which the true effect size in a new prevalence study will lie, were also computed and reported (41).

In addition to the overall weighted CD prevalence, the weighted CD prevalence were determined by analyzing subgroups according to TS type, sample size (<50 or ≥50 patients with TS), and CD diagnostic method (medical records, serology, biopsy, or unclear). Additionally, for each subgroup, the number of studies, number of patients with TS tested for CD, number of patients with TS diagnosed with CD, and median CD prevalence with ranges were also reported.

To determine the contribution of sample size and CD diagnostic method to the variability in CD prevalence rates across the studies, univariate and multivariate random-effects meta-regression models were performed. In the multivariate model, a *p-*value of ≤ 0.05 was considered indicative of statistical significance, which contributed to the heterogeneity in prevalence estimates. The number of studies in the reported subcategories was low; therefore, TS type was not used in the meta-regression analysis to preserve sufficient power.

### Risk of Bias Assessment

The risk of bias (RoB) of the reviewed individual studies was evaluated using six criteria related to prevalence studies included in the National Heart, Lung, and Blood Institute risk assessment tool (42). The six quality-related criteria assessed whether the study population was clearly specified, participation rate was at least 50%, justification for the recruited sample size was provided, all the participants were selected from the same or similar populations, and the outcome measure was clearly defined, valid, reliable, and implemented consistently across all study participants. The potential answer for each of these criteria was either “yes, no, or an unclear.” For additional quality assessment, we also determined the robustness of the implemented sampling methodology (probability-based, not probability-based, or unclear sampling methodology) as the seventh criterion. Studies were considered to be of high quality if patients with TS tested for CD were selected following probability-based sampling. In the event of insufficient information on any of the quality assessment criteria, the study was categorized as unclear. The overall proportion of individual studies with potentially low RoB across the seven quality criteria was determined. The mean study quality score was also computed based on a maximum quality score of seven.

Quality assessment was independently performed by two reviewers (GSM-AB and AH-N). Any disagreements between the reviewers in the extraction phase or during quality assessment were discussed and resolved by consensus.

### Publication Bias

A contour-enhanced funnel plot was constructed to explore the effects of small studies on the pooled CD prevalence. The funnel plot was constructed by plotting each CD prevalence measure against its standard error. Asymmetry of the funnel plot was tested using Egger's test (43).

The *metaprop* (44) and *metareg* packages of Stata v15 software (45) were used for analyses.

## Results

### Scope of the Review

**Figure 1:**
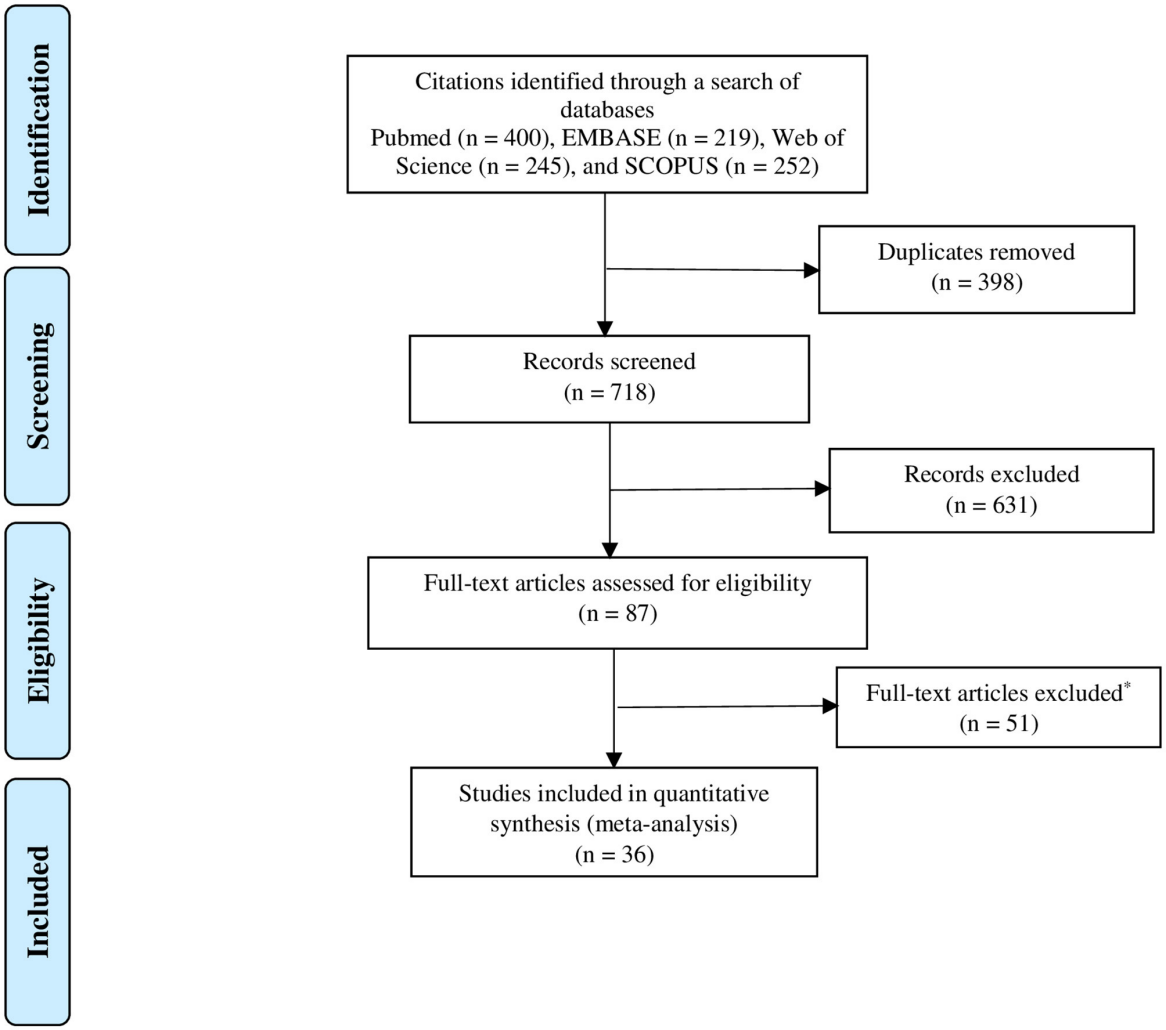
PRISMA flow chart of study selection.

Among a total of 1,116 citations retrieved from the four databases, 36 research articles that fulfilled the eligibility criteria were included in the quantitative meta-analysis (Figure 1). Table 1 summarizes descriptive information of the 36 research articles. These articles (29, 46–48, 50–80) were from 19 countries (Italy, Sweden, Canada, Poland, France, Iran, Germany, Denmark, The United Kingdom, Brazil, The United States of America, The Netherland, Ireland, Turkey, Czech Republic, Algeria, Tunisia, Romania, and Egypt), with the majority of the articles from Europe (47.2%) (29, 46, 47, 49–51, 53, 55, 56, 59, 61, 62, 64, 66, 67, 69, 71, 73, 76–78, 80), Canada (11.1%) (48, 60, 65, 68), and the United States of America (8.3%) (58, 60, 75). The predominantly used CD diagnostic method was biopsy in 55.6% of the research articles. These 36 research articles included 40 studies (single prevalence estimate) on CD prevalence in patients with TS. The TS type was specified in only two articles (53, 75), whereas all TS types were considered in 24 studies.

**Table 1:** Summary of studies reporting the prevalence of CD in patients with TS and/or association between TS and CD.

**Author, year**	**Duration of data collection**	**Country, city**	**Study design**	**Sampling method**	**Study population**	**Strata**	**TS type**	**CD diagnostic method**	**Sample size**	**Number of patients with CD**	**Prevalence (%)**	**Estimate of association**
Bonamico et al. (46)	–	Italy, Catania and Rome	Cross-sectional	Unclear	Patients with TS observed at pediatric clinics at the University of Rome and University of Catania	All	Unclear	Biopsy	37	3	8.1	–
Ivarsson et al. (47)	–	Sweden	Cross-sectional	Unclear	Patients with TS aged 3–16 years (mean age, 10 years) in a Swedish multicenter trial to promote growth in patients with TS	All	All types	Biopsy	87	4	4.6	–
Gillet et al. (48)	01/12/1998–01/10/1999	Canada, British Columbia	Cross-sectional	Whole population	Patients with TS followed up at the British Columbia's Children's Hospital	All	Unclear	Biopsy	45	1	2.2	–
Rujner et al. (49)	–	Poland, Warsaw	Cross-sectional	Unclear	Patients with TS who attended the Outpatient Department of Children's Memorial Health Institute in Warsaw	All	Unclear	Biopsy	48	2	4.2	–
Bonamico et al. (50)	–	Italy, various cities	Cross-sectional	Unclear	Patients with TS aged 7–38 years recruited from various centers in the Northern, Central, Southern, and Insular Italian regions	All	All types	Biopsy	389	25	6.4	–
Sakly et al. (51)	–	France, Lyon	Cross-sectional	Unclear	Patients from several Departments of Pediatrics over an 18-month period (Hospices Civils de Lyon, France)	All	Unclear	Sero anti-tTG Abs or AEA positive	47	7	14.9	–
Moayeri and Bahremand (52)	00/10/2002–2004	Iran, Tehran	Cross-sectional	Unclear	Patients with TS who attended the Pediatric Clinic at Tehran University of Medical Sciences	All	Unclear	Biopsy	48	2	4.2	–
Bettendorf et al. (53)	–	Germany	Cross-sectional	Unclear	Patients with TS aged >16 years from 96 German centers recorded until January 2000 in the IGLU database	TS Karyotype	All types	Sero anti-tTG Abs or AEA positive	120	5	4.2	–
							Classic		72	3	4.2	
							Mosaic		7	0	0.0	
							46, X, i (xq)		7	1	14.3	
							others		34	1	2.9	
Ságodi et al. (54)	1994–2003	–	–	Unclear	–	All	Unclear	Biopsy	63	5	7.9	–
Motenson et al. (55)	–	Denmark	Cross-sectional	Whole population	Danish patients with TS recruited from the National Society of Turner Contact Groups in Denmark, the Medical Department at Aarhus University Hospital, the Pediatric Unit at Hillerød Hospital, and Children's Hospital at Glostrup Hospital	All	All types	Biopsy	106	5	4.7	–
Frost et al. (56)	–	UK, London	Cross-sectional	Consecutive	Women with karyotypically proven TS who attended the Adult Turner Clinic at University College Hospital, London	All	All types	Biopsy	256	12	4.7	–
Dias et al. (57)	–	Brazil, Brasilia	Cross-sectional	Unclear	Patients with TS followed up at the Clinical Genetic Unit of Brasilia University Hospital	All	All types	Biopsy	56	2	3.6	–
Nabhan and Eugester (58)	00/00/2000–00/00/2010	USA, Indiana	Cross-sectional	Whole population	Girls followed up for TS at the Endocrine Clinic at Riley Hospital for Children in Indianapolis, Indiana	All	All types	Sero anti-tTG Abs or AEA positive	77	4	5.2	–
Freriks et al. (59)	00/05/2005–00/06/2009	Netherland, Nijmegen	Cross-sectional	Consecutive	Adult women with TS at a multidisciplinary care unit for adult women with TS	All	All types	Sero anti-tTG Abs or AEA positive	150	3	2.0	–
Bakalov et al. (60)	00/01/2000–00/03/2009	USA, Bethesda	Cross-sectional	Consecutive	Patients with TS at the Clinical Center of the National Institutes of Health (NIH) recruited primarily through notices on the internet and the NIH home page	All	All types	Medical records	224	6	2.7	RR, 42.5 (95% CI, 12.4–144.8)
Nadeem and Roche (61)	–	Ireland, Dublin	Cross-sectional	Unclear	Patients with TS who visited the Department of Pediatrics, University of Dublin	All	All types	Biopsy	32	3	9.4	–
Goldacre and Seminog (62)	1999–2011	UK, England	Retrospective	Unclear	A cohort of female patients hospitalized with TS	All	Unclear	Medical records	2,459	45	1.8	RR, 14 (95% CI, 1.48–12.75)
Yesilkaya et al. (63)	00/09/2013–31/01/2014	Turkey	Cross-sectional	Unclear	Patients with TS aged 0–18 years who were followed in 35 different centers in different regions of Turkey	All	All types	Biopsy	698	18	2.6	–
Rutigliano et al. (64)	–	Italy	Cross-sectional	Unclear	A cohort of 31 children with TS	All	All types	Medical records	31	4	12.9	–
Hirschfield et al. (65)	–	Canada, Ontario	Cross-sectional	Consecutive	Patients with TS aged 8–18 years enrolled from two pediatric TS clinics in Ontario	All	All types	Biopsy	63	4	6.3	–
Gawlik et al. (66)	–	Poland, Silesia	Case-control	Consecutive	Patients with TS treated at the Department of Pediatric Endocrinology	All	Unclear	Unclear	37	3	8.1	–
Stocklasova et al. (67)	–	Czech Republic	Cross-sectional	Unclear	A cohort of 286 Czech females with TS followed up at pediatric tertiary centers and later at adult tertiary centers in the Czech Republic	All	All types	Biopsy	286	25	8.7	–
Farquhar et al. (68)	01/02/2015–01/07/2018	Canada, Toronto	Cross-sectional	Whole population	Patients with TS evaluated at a multidisciplinary TS clinic at a university-based ambulatory hospital in Toronto	All	All Types	Medical records	122	11	9.0	–
						<40-years old			73	7	9.6	
						≥40-years old			49	4	8.2	
Wegiel et al. (69)	00/00/2001–00/00/2018	Poland, Silesia	Cross-sectional	Unclear	134 patients with TS treated at the Department of Pediatric Endocrinology	All	All types	Biopsy	73	2	2.7	–
Ouidad et al. (70)	2015–2017	Algeria, Algeria	Cross-sectional	Unclear	Children and adolescents with TS	All	All types	Biopsy	85	12	14.1	–
Stagi et al. (71)	06/2003–05/2011	Italy, Avellino and Florence	Prospective Cohort	Unclear	Patients with TS with a median age of 16.2 years	All	Unclear	Biopsy	32	3	9.4	OR, 18.1 (95% CI, 1.82–180)
Kammoun et al. (72)	01/2007–12/2011	Tunisia	Cross-sectional	Unclear	Patients with TS	All	All types	Unclear	37	2	5.4	–
Berglund et al. (73)	2003–2008	Denmark	Cross-sectional	Unclear	Girls and women with TS from the National Society of Turner Contact Groups in Denmark, Aarhus University Hospital, Hillerød Hospital, and Children's Hospital at Glostrup Hospital	All	All types	Biopsy	141	2	1.4	–
Bessahraoui et al. (74)	2007–2013	Algeria	Cross-sectional	Unclear	Children with TS observed over a 7-year period	All	All types	Unclear	33	4	12.1	–
Avolio et al. (75)	–	USA, Pittsburg	Cross-sectional	Unclear	Patients who presented at the Genetics and/or Endocrine clinic with varying mosaic TS karyotypes for evaluation	All	Mosaic	Medical records	40	1	2.5	–
Dumitrescu et al. (76)	–	Romania, Bucharest	Cross-sectional	Unclear	Girls diagnosed with TS at the C. I. Parhon National Institute of Endocrinology	All	All types	Unclear	93	3	3.2	–
Elechi et al. (77)	2008–2017	England, Nottingham	Cross-sectional	Unclear	Girls with TS who attended the over-12 TS clinic at Nottingham Children's Hospital	All	All types	Medical records	28	1	3.6	–
Grossi et al. (78)	–	Italy, Rome	Cross-sectional	consecutive	Patients with TS recruited from Bambino Gesu Children's Hospital in Rome	All	All types	Sero anti-tTG Abs	66	2	3.0	–
Hamza et al. (79)	00/10/2009–00/11/2010	Egypt, Cairo	Cross-sectional	Unclear	Patients with TS recruited from the Pediatric Endocrinology Clinic, Children's Hospital, Ain Shams University	All	All types	Biopsy	80	2	2.5	–
Marild et al. (29)	1997–2006	Sweden	Case-control	Whole population	Patients with TS registered in the National Patient Register	All	Unclear	Biopsy	5	1	20.0	OR: 4.34 (95% CI, 1.48–12.75)
Stenberg et al. (80)	–	Sweden, Stockholm	Cross-sectional	Unclear	Females with TS in the Stockholm area aged 7–65 years	All	All types	Medical records	97	4	4.1	–

*CI, confidence interval; TS, Turner syndrome; CD, celiac disease; tTG Abs, antibodies against tissue transglutaminase; AEA, anti-endomysium; IGLU, Internationale Genotropin Langzeit-Untersuchung; RR, rate ratio; OR, odds ratio*.

Only four research articles (29, 60, 62, 72) reported quantified or quantifiable information on the strength of association between TS and CD, with a heterogeneous study design and type of effect estimates.

### CD Prevalence in Patients With TS

The 40 studies that examined CD prevalence tested 6,291 patients with TS, yielding a crude CD prevalence of 3.8% (Table 2). The lowest CD prevalence of 0.0% was reported in a study of seven patients with mosaic TS in Germany (53), whereas the highest CD prevalence of 20.0% was reported in a study of 97 patients with TS registered in the National Patient Register in Sweden (80).

**Table 2:** Weighted prevalence of CD in patients with TS.

	**Number of studies**	**Number of patients tested for CD**	**CD**	**CD prevalence**				**Heterogeneity measures**		
				**Range (%)**	**Median (%)**	**Weighted prevalence (%)**	**95% CI**	**Q (*p*-value)^**a**^**	***I*^**2**^ (%)^**b**^**	**95% PI (%)^**c**^**
**TS type**										
Classical	1	72	3	4.2	–	–	–	–	–	–
Mosaic	2	47	1	0.0–2.5	1.25	0.7	0.0–7.5	–	–	–
46, X, I (Xq)	1	7	1	14.3	–	–	–	–	–	–
All types	24	3,244	161	1.4–14.1	4.9	4.2	1.4–11.5	53.2 (<0.001)	56.7	0.0–10.0
Unclear	12	2,921	75	1.8–20.0	6.0	4.9	3.7–6.4	37.2 (<0.001)	70.4	0.0–20.0
**Sample size**										
<50	18	597	43	0.0–20.0	8.1	5.9	3.9–8.3	14.2 (0.6)	0.0	0.0–10.0
≥50	22	5,694	198	1.4–14.1	4.4	4.4	3.1–5.8	83.0 (<0.001)	74.7	0.0–10.0
**CD diagnostic method**										
Medical records	7	2,970	68	1.8–9.6	3.6	3.6	1.6–6.3	18.4 (<0.001)	67.4	0.0–10.0
Serology	8	460	21	0.0–1.5	3.6	3.4	1.0–6.6	11.2 (0.1)	37.7	0.0–10.0
Biopsy	20	2,630	136	1.4–20.0	5.5	4.8	3.4–6.5	42.5 (<0.001)	55.3	0.0–10.0
Unclear	5	231	16	3.2–12.9	8.1	6.8	3.1–5.9	0.2 (<0.001)	26.2	1.0–20.0
Overall^d^	40	6,291	241	0.0–20.0	4.7	4.5	3.3–5.9	119.6 (<0.001)	67.4	0.0–10.0

*CD, celiac disease; TS, Turner syndrome; CI, confidence interval; PI, prediction interval*.

a *Q: Cochran's Q statistic is a measure assessing the existence of heterogeneity in estimates of CD prevalence*.

b *: a measure assessing the percentage of between-study variation due to differences in CD prevalence estimates across studies rather than chance*.

c *PI: estimates the 95% CI in which the true CD prevalence estimate in a new study is expected to fall*.

d *Overall pooled CD prevalence in patients with TS*.

**Figure 2:**
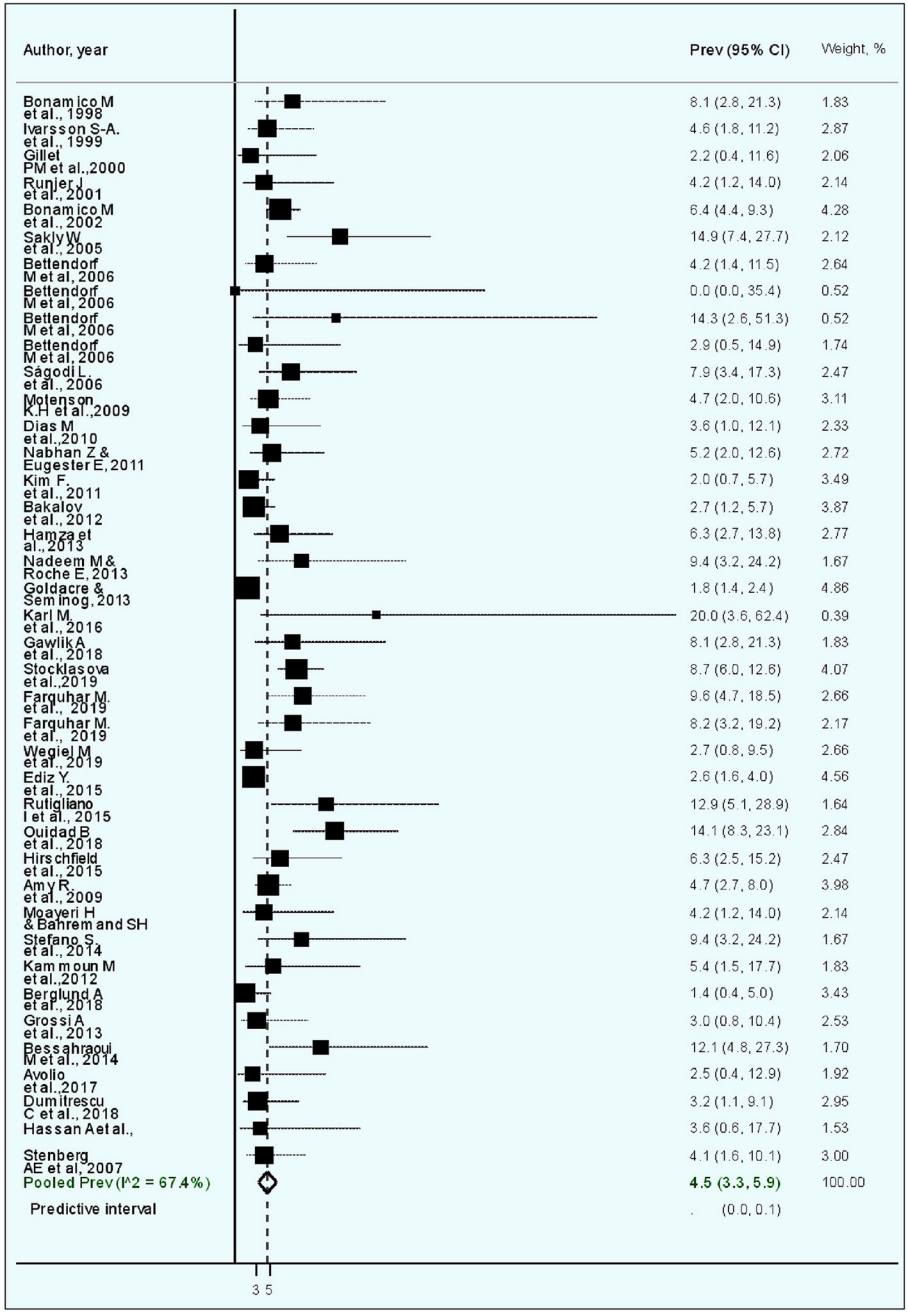
Forest plot of the meta-analysis of studies on celiac disease in patients with Turner syndrome. The diamond is centered on the summary prevalence estimate, and the width indicates the corresponding 95% confidence interval (CI).

The estimated weighted CD prevalence was 4.5% (95% CI, 3.3–5.9, *I*^2^, 67.4%; Table 2 and Figure 2). The weighted CD prevalence was similar between studies that included <50 patients with TS (5.9%, 95% CI, 3.9–8.3, *I*^2^, 0.0%) and those that included ≥50 patients with TS (4.4%, 95% CI, 3.1–5.8, *I*^2^, 74.7%; Table 2). The analysis according to the CD diagnostic methods used revealed that the highest estimated weighted CD prevalence was obtained from five studies including “unclear” as a diagnostic method (6.8%, 95% CI, 3.1–5.9, *I*^2^, 26.2%), followed by an estimated weighted CD prevalence of 4.8% (95% CI, 3.4–6.5, *I*^2^, 55.3%) obtained from 20 studies using biopsy. The 95% CI of the CD prevalence according to the four CD diagnostic method categories was overlapping (Table 2).

### Predictors of Heterogeneity in CD Prevalence

In the univariate meta-regression model, only sample size exhibited a significant association with variability in CD prevalence. The CD prevalence was 40% lower in studies that included ≥50 patients with TS (odds ratio 0.60, *p* = 0.022) than in studies that included <50 patients with TS. The observed significance in variability remained in the meta-regression model adjusted for the CD diagnostic method (adjusted odds ratio 0.61, 95% CI, 0.39–0.97; Table 3).

**Table 3:** Univariate and multivariable meta-regression analyses to identify the sources of heterogeneity in studies reporting the prevalence of CD in patients with Turner syndrome based on different characteristics.

	**Number of studies**	**OR (95% CI)**	***p-*value**	**aOR (95% CI)**	***p-*value**
**Sample size**			0.022		0.038
<50	18	1.00		1.00	
≥50	22	0.60 (0.39–0.92)		0.61 (0.39–0.97)	
**CD diagnostic method**					
Medical records	7	1.00		1.00	
Serology	8	1.31 (0.61–2.81)	0.474	1.31 (0.64–2.71)	0.452
Biopsy	20	1.41 (0.76–2.64)	0.269	1.47 (0.81–2.67)	0.199
Unclear	5	1.96 (0.85–4.52)	0.109	1.64 (0.73–3.69)	0.224

*OR, odds ratio; aOR, adjusted odds ratio; CI, confidence interval; CD, celiac disease*.

### Publication Bias in CD Prevalence

**Figure 3:**
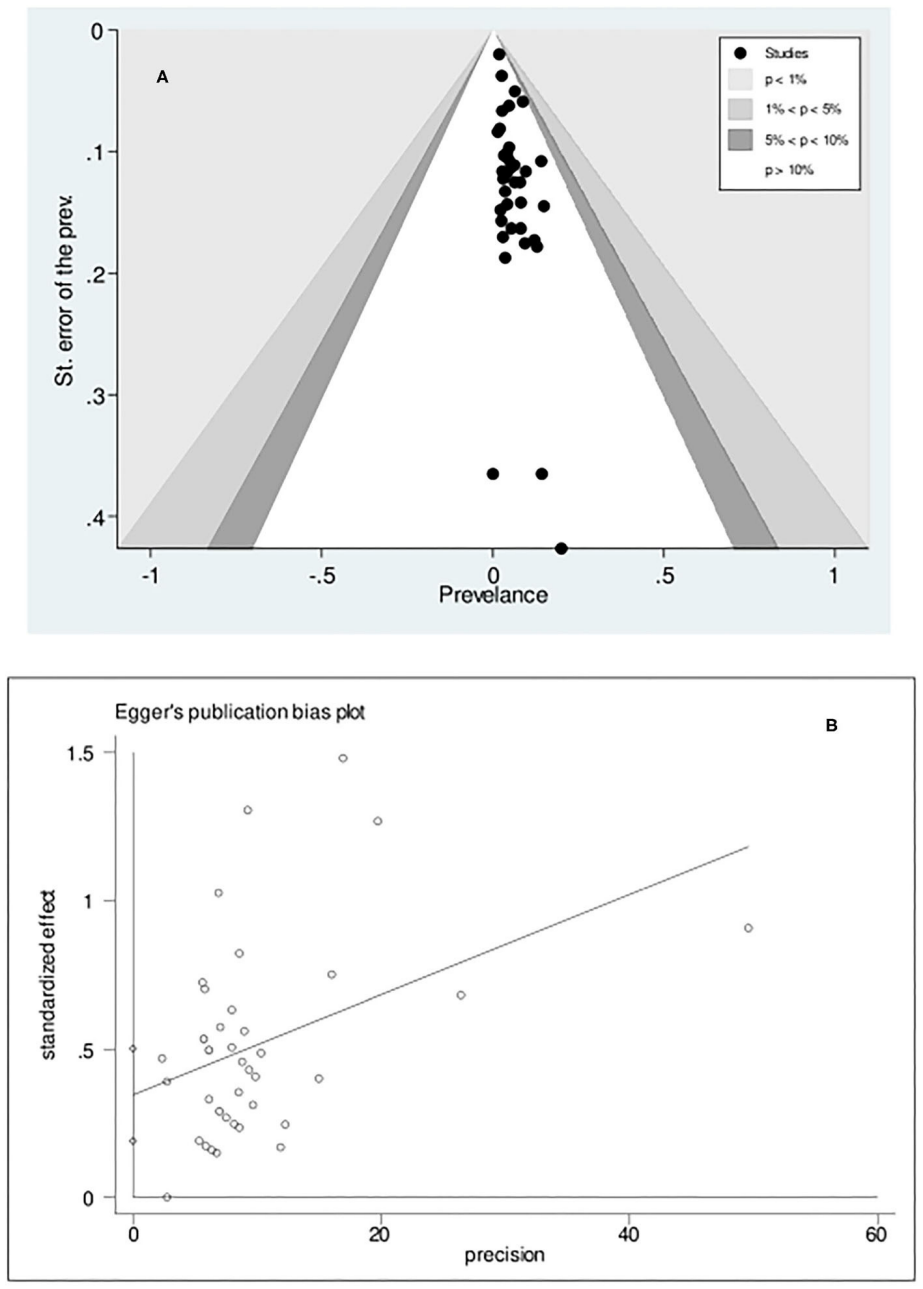
Contour-enhanced Funnel plot **(A)** and Egger's publication bias plot **(B)** examining small-study effects on the pooled celiac disease prevalence among patients with Turner syndrome. The estimated bias coefficient is 0.346 with a standard error of 0.077, indicating a *p-*value of < 0.001.

The statistical assessment (Egger's test, *p* < 0.001) of the funnel plot to determine the potential of publication bias due to the small-study effect suggested that there was asymmetry in the funnel plot, implicating the role of the small-study effect in CD prevalence (Figures 3A,B).

### Quality Assessment of the CD Prevalence

Supplementary Table 2 and Supplementary Figure 1 provide the details of RoB assessment using the seven assessment criteria. Briefly, the study population was clearly specified and defined in 72.2% of the reviewed articles, the selection of the TS population from the same or similar populations was clearly mentioned in 75.0% of the articles, and the outcome of CD was clearly defined in 83.3% of the articles. The sample size justification and power calculation were not reported in 86.1% of the articles. Overall, more than half (55.6%) of the 36 articles were deemed to have low RoB based on at least five of the seven RoB assessment criteria. Of a maximum score of 7, the mean RoB score was 3.8 for the 36 reviewed articles.

## Discussion

The present systematic review and meta-analysis summarized the burden of CD in patients with TS by evaluating its prevalence. The systematic review included 36 research articles yielding 40 prevalence studies that included a total of 6,291 patients with TS. The meta-analysis revealed that the CD prevalence was 4.5% in patients with TS. The estimated CD burden in patients with TS in the present meta-analysis is similar to that reported by other meta-analyses of different subject cohorts with chronic conditions or genetic disorders, including patients with type 1 diabetes mellitus (5%, 95% CI, 3–7) (81), iron deficiency anemia (3.2%, 95% CI, 2.6–3.9) (82), and irritable bowel syndrome (6.13%, 95% CI, 4.11–9.05) (83). The quantified weighted CD prevalence based on serology and biopsy (3.4 and 4.8%, respectively) in patients with TS is 2.4- and 6.4-fold higher, respectively, than the recently estimated pooled global (1.4%) CD prevalence in the general population (15) and higher than the biopsy-confirmed CD in general populations in several countries including Australia (0.46%) (84), Cuba (0.5%) (85), Finland (2.1%) (86), Iran (0.3%) (87), India (1.1%) (88), United Kingdom (0.6%) (89), and Germany (0.4%) (90). To the best of our knowledge, this is the first study to report the global pooled CD prevalence in patients with TS.

Previous studies have considered patients with TS as a group at risk of developing CD (30, 37, 91), and the present study provides evidence on the susceptibility of patients with TS to the development of CD. This finding is supported by evidence presented in three individual studies that reported an increased risk of CD in patients with TS (60, 62, 71). However, there was a lack of uniformity in the reported measures of association, including relative risk, risk ratio, and odds ratio, and study design. Therefore, producing a weighted measure of association was unfeasible. Moreover, the results of our meta-analysis support the findings of a review by Lleo et al. (92), who indicated that one of the most prominent characteristics of patients with TS was increased susceptibility to autoimmune diseases. Short stature in patients with TS has been related to genetic, skeletal, and growth hormone secretion abnormalities (93). Given these genetic abnormalities, there is an accumulative evidence on the increased susceptibility of patients with TS to develop autoimmune diseases (26, 94, 95) including CD and on the association between CD and other autoimmune diseases such as thyroiditis (28). Although the exact underlying pathophysiological mechanism between TS and CD is still unclear (55), humoral and cellular immune responses (96–99) as well as genetic contribution (100, 101) such as the alteration in the expression of the X-linked FoxP3 gene (102) have been suggested.

At the light of results of more prevalent CD in patients with TS compared to general populations, a comprehensive autoimmune screening would be advised in patients with TS syndrome assessing autoantibodies that can show associated autoimmune diseases/disorders in TS-CD patients (103–105). This supported by the recently published recommendations by the TS Consensus Group (32) that has specifically addressed the diagnostic screening process and the management of several comorbidities including CD in TS in the childhood (33). Moreover, given that cutaneous stigmata can provide critical clues for early detection of TS (106) and the high prevalence of CD in patients with atopic dermatitis (107), then patients with TS presented with atopic dermatitis or with cutaneous stigmata should be prioritized for the early screening and detection of CD. Additionally, since CD patients are also susceptible to neurological manifestations (108, 109), screening for the anti-neuronal antibodies is also recommended to be assessed in the work-up of patients with TS. A study suggested that CD in patients with TS is responsible for a failure of growth hormone therapy (46), hence early screening and management of CD in patients with TS could improve treatment outcomes and controlling for other comorbidities. Evidence-based guidelines in the management of not only CD but also other autoimmune disorders in patients with TS is warranted.

The strength of this study is the inclusion of studies from four large databases, which yielded a substantial sample size of patients with TS (*n* = 6,291) screened for CD. The review of the articles and the extraction of data by independent reviewers contributed to reducing the potential human error. Extracting and pooling stratified CD prevalence estimates as well as subgroup analyses according to the CD diagnostic methods also provided more stringent and potentially less biased prevalence estimates. A further strength of this study is the identification of gaps in evidence, specifically the lack of data on the burden of CD in patients with TS from several countries worldwide.

Conversely, we acknowledge some limitations that should be considered when interpreting the findings. First, there was lack of uniformity in the CD diagnostic methods among the studies, with the highest prevalence of CD reported in studies with no clear CD diagnostic method, which might have led to over- or under-estimation of CD prevalence. Second, most studies were from Western countries, which might affect the generalizability of the results at the regional, sub-regional, and global levels. Third, the publication bias assessment showed an asymmetry of the funnel plot, which might be a result of the small-study effect.

In conclusion, ~1 in 22 patients with TS had CD. Regular screening of patients with TS for CD will facilitate the early identification of asymptomatic CD, with early and better intervention ultimately leading to improvements in case management and health outcomes. Further studies are needed from countries that lack data on the burden of CD among various patient populations at risk for CD, including those with TS.

## Data Availability Statement

The raw data supporting the conclusions of this article will be made available by the authors upon justifiable requests.

## Author Contributions

RH-A performed data analysis and interpretation. GA-B and AA drafted the manuscript. RH-A and SA-S critically reviewed the drafted manuscript. All authors conceptualized the study objectives and design and reviewed and approved the final submitted version.

## Conflict of Interest

The authors declare that the research was conducted in the absence of any commercial or financial relationships that could be construed as a potential conflict of interest.

## Abbreviations

TS: Turner syndrome
CD, celiac disease, CI: confidence interval
RoB: Risk of bias.
