# Supplementary material for: Prevalence of Celiac Disease in Patients With Turner Syndrome: Systematic Review and Meta-Analysis
Source: Front Med (Lausanne). 2021 Jun 17;8:674896. doi: 10.3389/fmed.2021.674896 (PMC8247446; doi:10.3389/fmed.2021.674896)
Supplement: Supplementary file 1 [file Data_Sheet_1.pdf]

frontiers

Supplementary Material

Supplementary Table S1. PRISMA checklist

Section/topic	#	Checklist item	Reported on page #
**TITLE**			
Title	1	Identify the report as a systematic review, meta-analysis, or both<br>In the title, the report has been identified as a systematic review and meta-analysis	1
**ABSTRACT**			
Structured summary	2	Provide a structured summary including, as applicable: background; objectives; data sources; study eligibility criteria, participants, and interventions; study appraisal and synthesis methods; results; limitations; conclusions and implications of key findings; systematic review registration number.	1-2
**INTRODUCTION**			
Rationale	3	Describe the rationale for the review in the context of what is already known.	2
Objectives	4	Provide an explicit statement of questions being addressed with reference to participants, interventions, comparisons, outcomes, and study design (PICOS).	2
**METHODS**			
Protocol and registration	5	Indicate if a review protocol exists, if and where it can be accessed (e.g., Web address), and, if available, provide registration information including registration number.	3
Eligibility criteria	6	Specify study characteristics (e.g., PICOS, length of follow-up) and report characteristics (e.g., years considered, language, publication status) used as criteria for eligibility, giving rationale.	3
Information sources	7	Describe all information sources (e.g., databases with dates of coverage, contact with study authors to identify additional studies) in the search and date last searched.	3
Search	8	Present full electronic search strategy for at least one database, including any limits used, such that it could be repeated.	3-4
Study selection	9	State the process for selecting studies (i.e., screening, eligibility, included in systematic review, and, if applicable, included in the meta-analysis).	3-4
Data collection process	10	Describe method of data extraction from reports (e.g., piloted forms, independently, in duplicate) and any processes for obtaining and confirming data from investigators.	3-4
Data items	11	List and define all variables for which data were sought (e.g., PICOS, funding sources) and any assumptions and simplifications made.	3-4
Risk of bias in individual studies	12	Describe methods used for assessing risk of bias of individual studies (including specification of whether this was done at the study or outcome level), and how this information is to be used in any data synthesis.	4-5
Summary measures	13	State the principal summary measures (e.g., risk ratio, difference in means).	4

Synthesis of results	14	Describe the methods of handling data and combining results of studies, if done, including measures of consistency (e.g., I^2) for each meta-analysis.	4
Risk of bias across studies	15	Specify any assessment of risk of bias that may affect the cumulative evidence (e.g., publication bias, selective reporting within studies).	5
Additional analyses	16	Describe methods of additional analyses (e.g., sensitivity or subgroup analyses, meta-regression), if done, indicating which were prespecified.	4
**RESULTS**			
Study selection	17	Give numbers of studies screened, assessed for eligibility, and included in the review, with reasons for exclusions at each stage, ideally with a flow diagram.	5
Study characteristics	18	For each study, present characteristics for which data were extracted (e.g., study size, PICOS, follow-up period) and provide the citations.	5 and table 1
Risk of bias within studies	19	Present data on risk of bias of each study and, if available, any outcome level assessment (see item 12).	6
Results of individual studies	20	For all outcomes considered (benefits or harms), present, for each study: (a) simple summary data for each intervention group (b) effect estimates and confidence intervals, ideally with a forest plot.	4-6
Synthesis of results	21	Present results of each meta-analysis done, including confidence intervals and measures of consistency.	4-6
Risk of bias across studies	22	Present results of any assessment of risk of bias across studies (see Item 15).	4-6
Additional analysis	23	Give results of additional analyses, if done (e.g., sensitivity or subgroup analyses, meta-regression [see Item 16]).	4-6
**DISCUSSION**			
Summary of evidence	24	Summarize the main findings including the strength of evidence for each main outcome; consider their relevance to key groups (e.g., healthcare providers, users, and policy makers).	6
Limitations	25	Discuss limitations at study and outcome level (e.g., risk of bias), and at review-level (e.g., incomplete retrieval of identified research, reporting bias).	7
Conclusions	26	Provide a general interpretation of the results in the context of other evidence, and implications for future research.	7
**FUNDING**			
Funding	27	Describe sources of funding for the systematic review and other support (e.g., supply of data); role of funders for the systematic review.	7

Box 1. Database and predetermined search strategy

Source	Search string	Results	Notes
**PubMed**	(("autoimmune disease"[Title/Abstract] OR "Autoimmune Diseases"[Mesh:NoExp] OR "disease, autoimmune"[Title/Abstract] OR "diseases, autoimmune"[Title/Abstract] OR "autoimmune diseases"[Title/Abstract] OR "celiac disease"[Title/Abstract] OR "coeliac disease"[Title/Abstract] OR sprue[Title/Abstract] OR gluten*[Title/Abstract] OR antiendomysium [Title/Abstract] OR antiendomysial [Title/Abstract] OR antigliadin [Title/Abstract] OR antireticulin [Title/Abstract] OR "tissue transglutaminase" [Title/Abstract] OR endomysium [Title/Abstract] OR endomysial [Title/Abstract] OR gliadin [Title/Abstract] OR Reticulin [Title/Abstract] OR "Celiac Disease"[Mesh]) AND ("Chromosome Disorders"[Mesh:NoExp] OR "chromosomes abnormalities"[Title/Abstract] OR "chromosome disorders"[Title/Abstract] OR "chromosomal disorder"[Title/Abstract] OR "chromosomal disorders"[Title/Abstract] OR "chromosome abnormality"[Title/Abstract] OR "chromosome abnormalities"[Title/Abstract] OR "chromosomal abnormalities"[Title/Abstract] OR "chromosomal abnormality"[Title/Abstract] OR "chromosomes abnormality"[Title/Abstract] OR "chromosomes aberrations"[Title/Abstract] OR "chromosome aberrations"[Title/Abstract] OR "Chromosome Aberrations"[Mesh:NoExp] OR "chromosomes aberration"[Title/Abstract] OR "chromosome aberration"[Title/Abstract] OR "Turner Syndromes"[Title/Abstract] OR "Bonnevie-Ullrich Syndrome"[Title/Abstract] OR "Turner's Syndrome*"[Title/Abstract] OR "Turners Syndrome"[Title/Abstract] OR "Gonadal Dysgenesis, 45,X"[Title/Abstract] OR "XO Gonadal Dysgenesis"[Title/Abstract] OR "Monosomy X"[Title/Abstract] OR "Status Bonnevie-Ullrich"[Title/Abstract] OR "Turner Syndrome"[Mesh] OR "Turner Syndrome"[Title/Abstract] OR "Ullrich-Turner Syndrome"[Title/Abstract]))	**400**	All search terms are searched in the search fields: “title” and “abstract” (here marked with TI/AB) and in MeSH (when available).<br><br>Filter for publication year from 01/01/1991 to 12/31/2019
**Web of Science** (Core Collection)	((TOPIC: "Turner Syndromes" OR "Bonnevie-Ullrich Syndrome" OR "Turner's Syndrome" OR "Turners Syndrome" OR "Gonadal Dysgenesis, 45,X" OR "XO Gonadal Dysgenesis" OR "Monosomy X" OR "Status Bonnevie-Ullrich" OR "Turner Syndrome" OR "Ullrich-Turner Syndrome" OR "chromosomes aberrations" OR "chromosome aberrations" OR "chromosomes aberration" OR "chromosome aberration" OR "chromosomes abnormalities" OR "chromosome disorders" OR "chromosomal disorder" OR "chromosomal disorders" OR "chromosome abnormality" OR "chromosome abnormalities" OR "chromosomal abnormalities" OR "chromosomal abnormality" OR "chromosomes abnormality") AND (TOPIC: "autoimmune disease" OR "disease, autoimmune" OR "diseases, autoimmune" OR "autoimmune diseases" OR "celiac disease" OR "coeliac disease" OR sprue OR gluten* OR antiendomysium OR antiendomysial OR antigliadin OR antireticulin OR "tissue transglutaminase" OR endomysium OR endomysial OR gliadin OR reticulin))	**245**	All search terms are searched in the field: “topic” (including title, abstract and author supplied keywords, here marked with “TOPIC”).<br><br>No thesaurus available.<br><br>Filter for publication year from 01/01/1991 to 12/31/2019
**Scopus**	((TITLE-ABS("Turner Syndromes" OR "Bonnevie-Ullrich Syndrome" OR "Turner's Syndrome" OR "Turners Syndrome" OR "Gonadal Dysgenesis, 45,X" OR "XO Gonadal Dysgenesis" OR "Monosomy	**257**	All search terms are searched in the search fields:

	X" OR "Status Bonnevie-Ullrich" OR "Turner Syndrome" OR "Ullrich-Turner Syndrome" OR "chromosomes aberrations" OR "chromosome aberrations" OR "chromosomes aberration" OR "chromosome aberration" OR "chromosomes abnormalities" OR "chromosome disorders" OR "chromosomal disorder" OR "chromosomal disorders" OR "chromosome abnormality" OR "chromosome abnormalities" OR "chromosomal abnormalities" OR "chromosomal abnormality" OR "chromosomes abnormality") AND (TITLE-ABS ("autoimmune disease" OR "disease, autoimmune" OR "diseases, autoimmune" OR "autoimmune diseases" OR "celiac disease" OR "coeliac disease" OR sprue OR gluten* OR antiendomysium OR antiendomysial OR antigliadin OR antireticulin OR "tissue transglutaminase" OR endomysium OR endomysial OR gliadin OR reticulin))		"title and "abstract" (here marked with "TITLE-ABS")<br><br>No thesaurus available.<br><br>Filter for publication year from 01/01/1991 to 12/31/2019
**Embase**<br><br>**Source**: "Embase Only" and "Embase and Medline"	(('chromosome disorders'/mj OR 'chromosomes abnormalities':ab,ti OR 'chromosome disorders':ab,ti OR 'chromosomal disorder':ab,ti OR 'chromosomal disorders':ab,ti OR 'chromosome abnormality':ab,ti OR 'chromosome abnormalities':ab,ti OR 'chromosomal abnormalities':ab,ti OR 'chromosomal abnormality':ab,ti OR 'chromosomes abnormality':ab,ti OR 'chromosomes aberrations':ab,ti OR 'chromosome aberrations':ab,ti OR 'chromosome aberrations'/mj OR 'chromosomes aberration':ab,ti OR 'chromosome aberration':ab,ti OR'turner syndromes':ab,ti OR 'bonnevie-ullrich syndrome':ab,ti OR 'turners syndrome':ab,ti OR 'gonadal dysgenesis, 45,x':ab,ti OR 'xo gonadal dysgenesis':ab,ti OR 'monosomy x':ab,ti OR 'status bonnevie-ullrich':ab,ti OR 'turner syndrome'/exp OR 'turner syndrome':ab,ti OR 'ullrich-turner syndrome':ab,ti) AND ('autoimmune disease':ab,ti OR 'autoimmune diseases'/mj OR 'disease, autoimmune':ab,ti OR 'diseases, autoimmune':ab,ti OR 'autoimmune diseases':ab,ti OR 'celiac disease':ab,ti OR 'coeliac disease':ab,ti OR sprue:ab,ti OR gluten*:ab,ti OR antiendomysium:ab,ti OR antiendomysial:ab,ti OR antigliadin:ab,ti OR antireticulin:ab,ti OR 'tissue transglutaminase':ab,ti OR endomysium:ab,ti OR endomysial:ab,ti OR gliadin:ab,ti OR reticulin:ab,ti OR 'celiac disease'/exp))	**219**	All search terms are searched in the fields: "title" and "abstract" (here marked with ":ab,ti") and in the "thesaurus" (here marked with "/de") when available.<br><br>Filter for publication year from 01/01/1991 to 12/31/2019

Supplementary Table S2. Quality assessment of the reviewed 36 published articles on celiac disease prevalence among patients with Turner syndrome

Author, year	RoB assessment criteria							Low RoB*
	Criterion #1	Criterion #2	Criterion #3	Criterion#4	Criterion #5	Criterion #6	Criterion #7	
Bonamico M. et al., 1998	1	1	1	1	2	1	3	5
Ivarsson S-A. et al., 1999	1	1	1	1	2	1	3	5
Gillet P.M. et al., 2000	1	3	1	1	2	1	1	5
Rujner J. et al., 2001	1	3	1	1	2	1	3	4
Bonamico M. et al., 2002	1	1	1	1	2	1	3	5
Sakly W. et al., 2005	1	1	1	2	2	1	3	4
Bettendorf M. et al, 2006	1	1	1	1	2	1	3	5
Ságodi L. et al., 2006	3	3	3	3	2	3	3	0
Motenson K.H. et al., 2009	1	1	1	1	2	1	1	6
Dias M. et al., 2010	1	3	1	1	2	1	3	4
Nabhan Z. & Eugester E, 2011	1	3	1	1	3	1	1	5
Freriks K. et al., 2011	1	1	1	1	2	1	1	6
Bakalov et al., 2012	1	1	1	1	3	1	1	6
Nadeem M. & Roche E., 2013	3	1	3	3	2	3	3	1
Goldacre M. & Seminog O., 2013	1	1	1	1	3	1	3	5
Gawlik T. et al., 2018	1	1	1	1	2	3	1	5
Stocklasova et al., 2019	1	3	2	3	2	1	3	2
Farquhar M. et al., 2019	1	1	1	3	2	1	1	5

Wegiel M. et al., 2019	1	1	1	1	2	1	3	5
Yesilkaya E. et al., 2015	1	1	1	1	2	1	3	5
Rutigliano I. et al., 2015	3	3	3	3	2	1	3	1
Ouidad B. et al., 2018	3	3	3	3	2	1	3	1
Hirschfield et al., 2015	1	1	1	1	2	1	1	6
Frost R. et al., 2009	1	1	1	1	2	1	1	6
Moayeri H. & Bahremand S.H., 2005	1	3	1	1	2	1	3	4
Stagi S. et al., 2014	2	1	3	3	2	1	3	2
Kammoun M. et al., 2012	3	3	3	3	3	3	3	0
Berglund A. et al., 2018	2	3	1	3	2	1	3	2
Bessahraoui M. et al., 2014	3	3	3	3	2	3	3	0
Avolio et al., 2017	3	3	3	3	2	1	3	1
Dumitrescu C. et al., 2018	3	3	1	3	2	3	3	1
Elechi A. et al., 2018	1	3	1	3	2	1	3	3
Grossi A. et al., 2013	1	1	1	1	2	1	1	5
Hamza T. et al., 2013	1	1	1	1	2	1	3	5
Marild K. et al., 2016	1	3	1	1	1	1	1	6
Stenberg A.E. et al, 2007	1	1	1	1	2	1	3	5
Mean quality score out of 7								**3.8**

RoB, risk of bias
* Total number of criteria assessed as with "low RoB" for each individual study

Criterion #1: Was the study population clearly specified and defined? Potential answer (1, Yes; 2, No; 3, Unclear).

Criterion #2: Was the participation rate of eligible persons at least 50%? Potential answer (1, Yes; 2, No; 3, Unclear).

Criterion #3: Were all the participants selected or recruited from the same or similar populations (including the same time period)? Potential answer (1, Yes; 2, No; 3, Unclear).

Criterion #4: Were the inclusion and exclusion criteria for participating in the study prespecified and applied uniformly to all participants? Potential answer (1, Yes; 2, No; 3, Unclear).

Criterion #5: Was the sample size justification, power description, or variance and effect estimates provided? Potential answer (1, Yes; 2, No; 3, Unclear).

Criterion #6: Were the outcome measures (dependent variables) clearly defined, valid, reliable, and implemented consistently across all study participants? Potential answer (1, Yes; 2, No; 3, Unclear).

Criterion #7: Sampling methodology. Potential answer (1, probability-based “random, consecutive, or whole population within a specified period of time”; 2, non-probability based; 3, unclear).

Supplementary Figure S1. RoB assessment of the 36 reviewed articles reporting prevalence estimates of celiac disease among patients with Turner syndrome

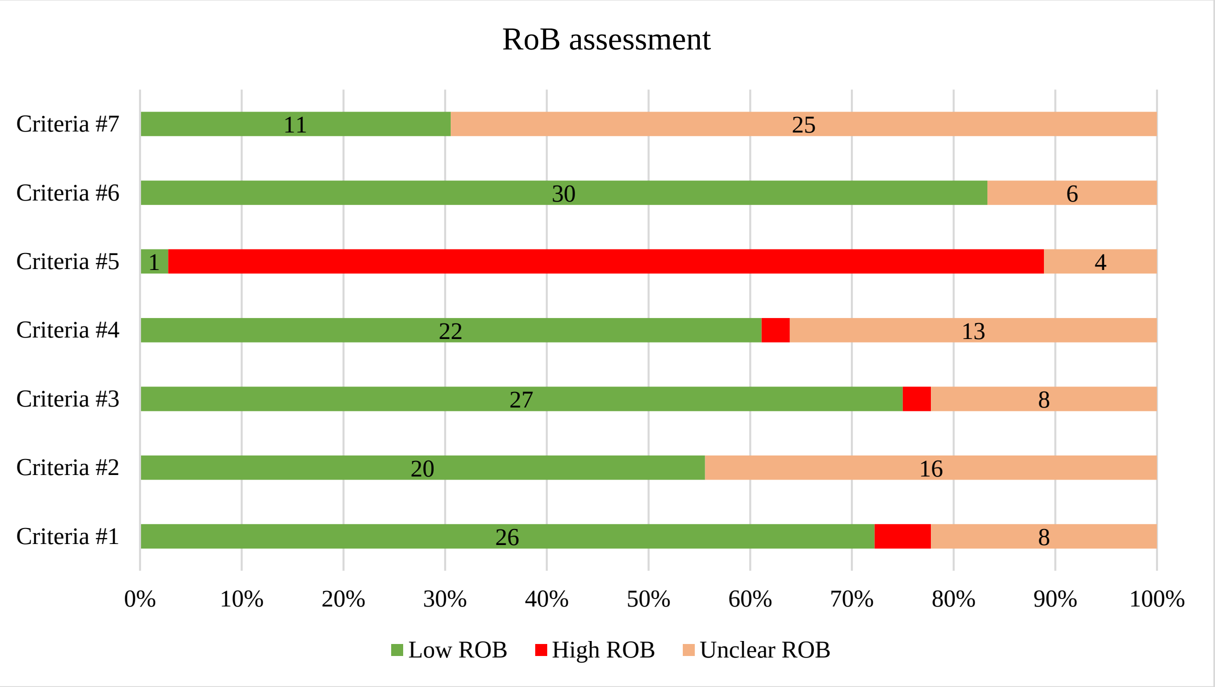

RoB, risk of bias

Criterion #1: Was the study population clearly specified and defined? Potential answer (1, Yes; 2, No; 3, Unclear).

Criterion #2: Was the participation rate of eligible persons at least 50%? Potential answer (1, Yes; 2, No; 3, Unclear).

Criterion #3: Were all the participants selected or recruited from the same or similar populations (including the same time period)? Potential answer (1, Yes; 2, No; 3, Unclear).

Criterion #4: Were the inclusion and exclusion criteria for participating in the study prespecified and applied uniformly to all participants? Potential answer (1, Yes; 2, No; 3, Unclear).

Criterion #5: Was the sample size justification, power description, or variance and effect estimates provided? Potential answer (1, Yes; 2, No; 3, Unclear).

Criterion #6: Were the outcome measures (dependent variables) clearly defined, valid, reliable, and implemented consistently across all study participants? Potential answer (1, Yes; 2, No; 3, Unclear).

Criterion #7: Sampling methodology. Potential answer (1, probability-based "random, consecutive, or whole population within a specified period of time"; 2, non-probability based; 3, unclear).
